# Artificial light at night: an underappreciated effect on phenology of deciduous woody plants

**DOI:** 10.1093/pnasnexus/pgac046

**Published:** 2022-04-18

**Authors:** Lin Meng, Yuyu Zhou, Miguel O Román, Eleanor C Stokes, Zhuosen Wang, Ghassem R Asrar, Jiafu Mao, Andrew D Richardson, Lianhong Gu, Yiming Wang

**Affiliations:** Department of Geological and Atmospheric Sciences, Iowa State University, Ames, IA 50010, USA; Department of Geological and Atmospheric Sciences, Iowa State University, Ames, IA 50010, USA; Universities Space Research Association, Columbia, MD 21046, USA; Universities Space Research Association, Columbia, MD 21046, USA; NASA Goddard Space Flight Center, Greenbelt, MD 20771, USA; NASA Goddard Space Flight Center, Greenbelt, MD 20771, USA; Earth System Science Interdisciplinary Center, University of Maryland, College Park, MD 20742, USA; Universities Space Research Association, Columbia, MD 21046, USA; Environmental Sciences Division and Climate Change Science Institute, Oak Ridge National Laboratory, Oak Ridge, TN 37831, USA; School of Informatics, Computing and Cyber Systems, Northern Arizona University, Flagstaff, AZ 86011, USA; Center for Ecosystem Science and Society, Northern Arizona University, Flagstaff, AZ 86011, USA; Environmental Sciences Division and Climate Change Science Institute, Oak Ridge National Laboratory, Oak Ridge, TN 37831, USA; Department of Geological and Atmospheric Sciences, Iowa State University, Ames, IA 50010, USA

**Keywords:** artificial light at night, phenology, light pollution, urbanization, climate change

## Abstract

Artificial light at night (ALAN), an increasing anthropogenic driver, is widespread and shows rapid expansion with potential adverse impact on the terrestrial ecosystem. However, whether and to what extent does ALAN affect plant phenology, a critical factor influencing the timing of terrestrial ecosystem processes, remains unexplored due to limited ALAN observation. Here, we used the Black Marble ALAN product and phenology observations from USA National Phenology Network to investigate the impact of ALAN on deciduous woody plants phenology in the conterminous United States. We found that (1) ALAN significantly advanced the date of breaking leaf buds by 8.9 ± 6.9 days (mean ± SD) and delayed the coloring of leaves by 6.0 ± 11.9 days on average; (2) the magnitude of phenological changes was significantly correlated with the intensity of ALAN (*P* < 0.001); and (3) there was an interaction between ALAN and temperature on the coloring of leaves, but not on breaking leaf buds. We further showed that under future climate warming scenarios, ALAN will accelerate the advance in breaking leaf buds but exert a more complex effect on the coloring of leaves. This study suggests intensified ALAN may have far-reaching but underappreciated consequences in disrupting key ecosystem functions and services, which requires an interdisciplinary approach to investigate. Developing lighting strategies that minimize the impact of ALAN on ecosystems, especially those embedded and surrounding major cities, is challenging but must be pursued.

Significance Statement ALAN profoundly disturbs the natural cycles of light and darkness that plants rely on to leaf out. In this study, we provide direct observational evidence that ALAN, an increasing environmental factor as a result of urbanization, advanced the date of breaking leaf buds and delayed the coloring of leaves in the conterminous United States. In a warmer and brighter night future, breaking leaf buds will continue to shift earlier, but the coloring of leaves will show a more complex response. The findings imply significant but underappreciated consequences of ALAN on terrestrial ecosystems, which requires an interdisciplinary approach to investigate.

## Introduction

Plants use natural light as an environmental cue on location, the time of day and year, and the surrounding environment (1). Photoperiod (i.e. daylength) determined by the natural cycles of light and darkness provides a consistent and reliable signal for plants to initiate seasonal phenological stages, such as leaf-out and flowering (2–6). However, the photoperiodic cue for plants has been profoundly disturbed by artificial light at night (ALAN) (7). Urbanization, electrification, population growth, and socio-economic development together cause an extensive expansion of ALAN in terms of both density and spatiotemporal distribution (8). ALAN exposure occurs widely over 23% of the global land surfaces between 75°N and 60°S and about 50% of the conterminous United States (9), even including the most protected habitats (10). More importantly, ALAN has rapidly increased by about 1.8% per year worldwide from 2012 to 2016, and this increasing trend is likely to continue in the future (8). Nevertheless, the consequences of ALAN on plant phenology are underappreciated (11). The lack of understanding on ALAN effect may lead to considerable uncertainties in the projection of future changes in phenology under urbanization and climate change, and would hinder a comprehensive understanding of anthropogenic influences on terrestrial ecosystems (12).

ALAN may influence phenology by changing plant perception of daylength and disturbing the circadian rhythms of plants (13). Evidence from manipulative laboratory experiments showed a wide range of plant responses to ALAN, including promotion of flowering and enhanced vegetative growth, even at low-intensity (7, 14–16). In the natural setting, earlier spring budburst (up to 7.5 days) (17) and later autumn senescence (by 13 to 22 days) (18, 19) were found in areas exposed to ALAN using satellite observations. Besides being affected by direct light in the illuminated urban area (e.g. domestic, architectural, advertising, and public street lightings) (20), plant phenology may also potentially be affected by low-intensity diffuse light from sky glow or transient light (e.g. vehicle lights) (21). The latter occurs over much larger areas surrounding cities and transportation network (21). Additionally, since plants mainly sense red and far-red light to constrain the circadian clock (22), lightbulb types that emit light at longer wavelengths (i.e. street lighting) are more effective in triggering phenological changes than the natural broad-spectrum white light (e.g. LED light) (7). However, these investigations on ALAN effects at the local scale have not been tested for large areas, i.e. entire ecosystems and ecoregion, due to the lack of ground observations and large uncertainty of remotely sensed ALAN observations. Moreover, the effect of urban heat island (i.e. elevated temperature in urban as compared to the rural areas) on phenology, which often accompanies ALAN in urban settings, was not rigorously considered in previous studies that investigated ALAN effect on phenology (17–19).

The NASA Black Marble ALAN products (23) provide a unique opportunity to explore the effect of ALAN on plant phenology at a global scale. Since the launch of Suomi-National Polar-orbiting Partnership (S-NPP) in 2011 and NOAA-20 in 2017, the Day/Night Band (DNB) sensors of the Visible Infrared Imaging Radiometer Suite (VIIRS), onboard these satellites, resulted in the first-ever high-resolution and well-calibrated low light imaging data of Earth's land area at night (23, 24). The light spectrum (i.e. 0.5 to 0.9 μm) that VIIRS DNB is sensitive to spans into the near-infrared region, which is critical to plant phenology. The main aim of this study is to use this new ALAN product to investigate the influence of ALAN on phenology of deciduous woody plants in the conterminous United States by addressing the following questions: (1) Whether and to what extent ALAN affects spring and autumn phenology, respectively? (2) Are there interactions between ALAN and temperature effects on the phenology? (3) How will phenology change in a warmer and brighter night future?

**Fig. 1.**
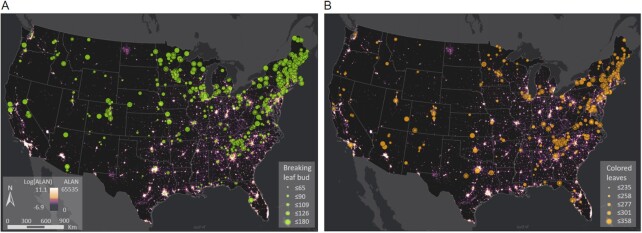
Spatial patterns of the breaking leaf buds (A) and colored leaves dates (B) under ALAN. The breaking leaf buds and colored leaves (day of year) obtained from the USA National Phenology Network are shown by the size of colored points. The background color represents ALAN intensity (nW/cm^2^/sr).

We focused our analysis on two phenological stages, breaking leaf buds in spring and colored leaves in autumn, obtained from the USA National Phenology Network dataset in the conterminous United States during 2011 to 2016 (Fig. 1; Table S1, Supplementary Material). We examined the differences in phenology at sites with and without ALAN while controlling temperature using the VIIRS Black Marble DNB ALAN product (Figure S1, Supplementary Material) and “Topography Weather” (TopoWx) climate dataset. We further investigated the confounding effects of temperature and ALAN on phenology with the consideration of species effect using a linear mixed model. Finally, we estimated phenological changes by the year 2100 under increasing ALAN and climate warming, using temperature simulations from 24 climate models of the sixth phase of the Coupled Model Intercomparison Project (CMIP6).

## Results

**Fig. 2.**
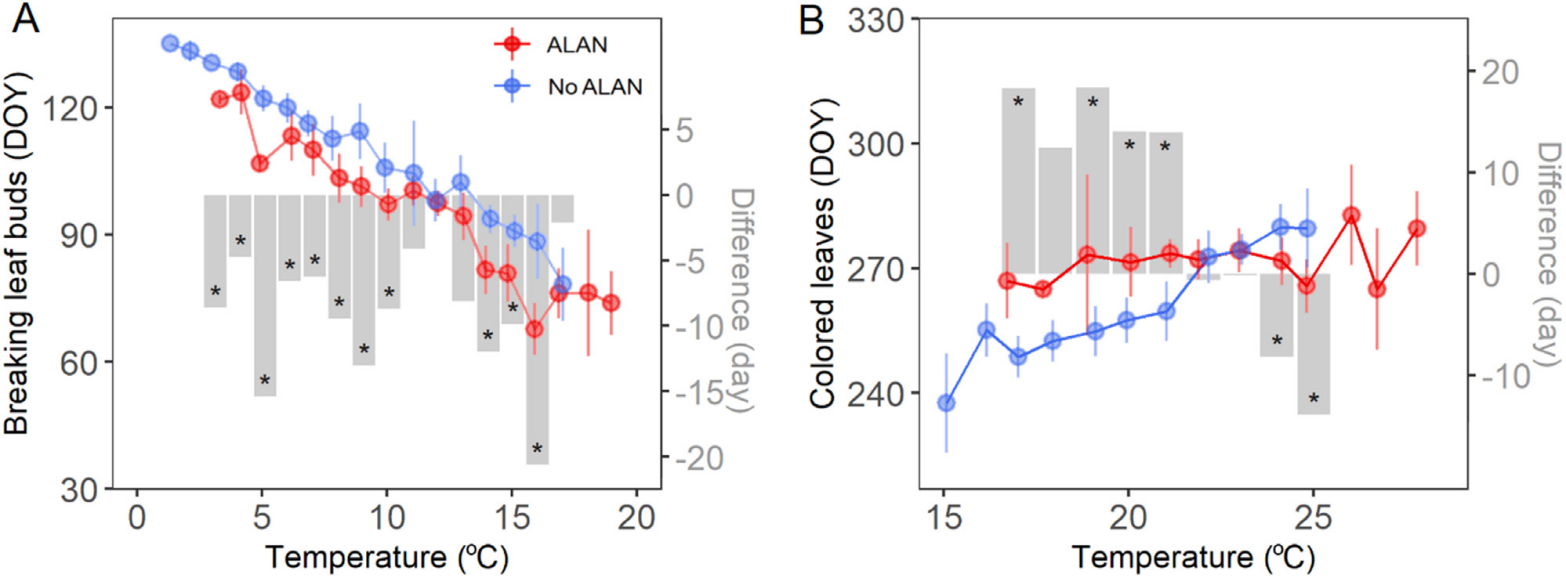
Differences in breaking leaf buds (A) and colored leaves (B) for sites with ALAN versus those without ALAN across temperatures. Points and error bars represent the mean and 95% CI of phenology (day of year) for each 1°C temperature increment. Gray bars represent the differences in phenology (days) by subtracting phenology at sites without ALAN from sites with ALAN. Statistical significance level at *P* < 0.05 is shown as an asterisk. The ALAN sites are those with higher than 75% quantile of ALAN among all sites (i.e. 19.1 nW/cm^2^/sr). Results based on bootstrapping method and alternative temperatures are shown in Figures S2 and S3 (Supplementary Material), respectively.

We found ALAN significantly shifted phenology by comparing phenology at sites with and without ALAN for every 1°C change in temperature (Fig. 2). In 11 out of 15 (i.e. 73.3%) temperature groups, breaking leaf buds were significantly earlier at sites with ALAN (sites with > 75% quantiles of ALAN) compared to sites with the same temperature but without ALAN (*P* < 0.05, Fig. 2A). Across all temperature groups, breaking leaf buds were on average 8.9 ± 6.9 days (mean ± SD) earlier at sites with ALAN (97.2 ± 14.6 day of year) than sites without ALAN (106.1 ± 14.1 day of year; Fig. 2A). The most considerable phenological difference was 19.8 days (*P* < 0.05), which occurred for the temperature group of 16°C. The magnitude of difference in breaking leaf buds between ALAN and non-ALAN sites (shown as gray bars) showed no correlation with temperature (*P* = 0.88, Fig. 2A). In contrast, ALAN-induced changes in colored leaves largely depend on temperature: colored leaves were delayed (on average 15.4 ± 2.8 days) when temperature was ≤ 21°C but advanced (on average 5.7 ± 6.6 days) when temperature was ≥ 22°C at sites with ALAN, compared to sites without ALAN (Fig. 2B). On average, colored leaves were delayed 6.0 ± 11.9 days at sites with ALAN (270.4 ± 3.6 day of year) than those without ALAN (264.4 ± 12.2 day of year) across all temperature groups (Fig. 2B). The magnitude of ALAN-induced difference in colored leaves was significantly correlated with temperature (reduced 4.0 days with 1°C warming, *P* < 0.001, Fig. 2B). Similar results were obtained after removing the influence of extreme climate and outlier phenological observation using a bootstrapping approach (Figure S2, Supplementary Material). We also examined ALAN-induced phenological changes using alternative temperature periods, i.e. February 1st to May 31st, January 1st to May 31st, and December 1st to May 31st for breaking leaf buds, and June 1st to September 31st, June 1st to October 31st, and June 1st to November 31st for colored leaves. We found consistent results although with different amplitudes (*P* < 0.05; Figure S3, Supplementary Material).

The ALAN effect on phenological changes was consistent across species though varied in magnitude (Figures S4 to S6, Supplementary Material). The top 10 species with the most observations all showed earlier breaking leaf buds at ALAN sites, compared to sites with a similar temperature but without ALAN (the difference in breaking leaf buds was significant for 6 out of 10 species, *P* < 0.05; Figures S4 and S6A, Supplementary Material). In 6 out of these 10 species, the magnitude of changes in breaking leaf buds ranged between 6 and 15 days (Figure S4, Supplementary Material). For the top eight species with the most observations of colored leaves, all species except for *Quercus alba* showed delayed dates at sites with ALAN than those without ALAN under similar temperature (Figure S5, Supplementary Material). On average, colored leaves were 11.2 ± 10.8 days later at sites with ALAN than those without ALAN (Figure S6B, Supplementary Material). Previous studies showed early species responded more strongly to warming than later species (25, 26), but we did not find such interspecies responses to ALAN, i.e. ALAN-driven phenological difference was not correlated with mean phenology across species for either breaking leaf buds (*P* = 0.67) and colored leaves (*P* = 0.25; Figure S6, Supplementary Material).

Both warmer temperature and brighter ALAN significantly advanced breaking leaf buds and delayed colored leaves (*P* < 0.001, Table 1). Specifically, an increase in 1°C in temperature on average advanced breaking leaf buds by 3.40 days (range of 3.18 to 3.61 days, with 97.5% CI) but delayed colored leaves by 1.30 (0.71 to 1.87) days, across all species for the mean ALAN level (*P* < 0.001, Table 1; Figure S7, Supplementary Material). An increase in 1 logarithm unit of ALAN (i.e. 172% increase in ALAN) on average advanced breaking leaf buds by 1.59 (1.18 to 2.01) days and delayed colored leaves by 2.60 (1.87 to 3.33) days across all deciduous woody plant species studied and at mean temperature level (*P* < 0.001, Table 1). In addition, we found the effect of species (SD of phenology explained by species: 7.55 days) was 3.6 times larger than the effect of year (SD of phenology explained by year: 2.09 days) on breaking leaf buds (Table S2, Supplementary Material). For colored leaves, 8.22 days variation was explained by the species effect, but only 0.51 days variation was explained by the year effect (Table S3, Supplementary Material). These results for the entire conterminous United States are consistent with previous studies at smaller scales, which showed earlier spring budburst in the United Kingdom (17) and delayed leaf fall in Slovakia (19) under increasing ALAN.

**Table 1.** Summary of linear mixed models for breaking leaf buds and colored leaves (day of year) stages. Linear mixed models include the fixed effects (temperature, log (ALAN), and the interaction term of temperature and ALAN, shown below) and random effects (species and year) for the intercept (shown as Tables S2 and S3, Supplementary Material). CI is 97.5%. The Estimates columns represent changes in phenology under 1°C increase in temperature or 1 unit of logarithmic increase in ALAN. The negative sign indicates phenology advance in days.

	Breaking leaf buds			Colored leaves		
Predictors	Estimates	CI	*P*	Estimates	CI	*P*
(Intercept)	0.33	−3.90 to 4.57	0.88	−0.73	−4.82 to 3.37	0.73
Temperature	−3.49	−3.69 to −3.29	**< 0.001**	1.30	0.71 to 1.87	**< 0.001**
Log (ALAN)	−1.43	−1.82 to −1.04	**< 0.001**	2.60	1.87 to 3.33	**< 0.001**
Interaction	0.02	−0.07 to 0.11	0.66	−0.59	−0.86 to −0.32	**< 0.001**

**Fig. 3.**
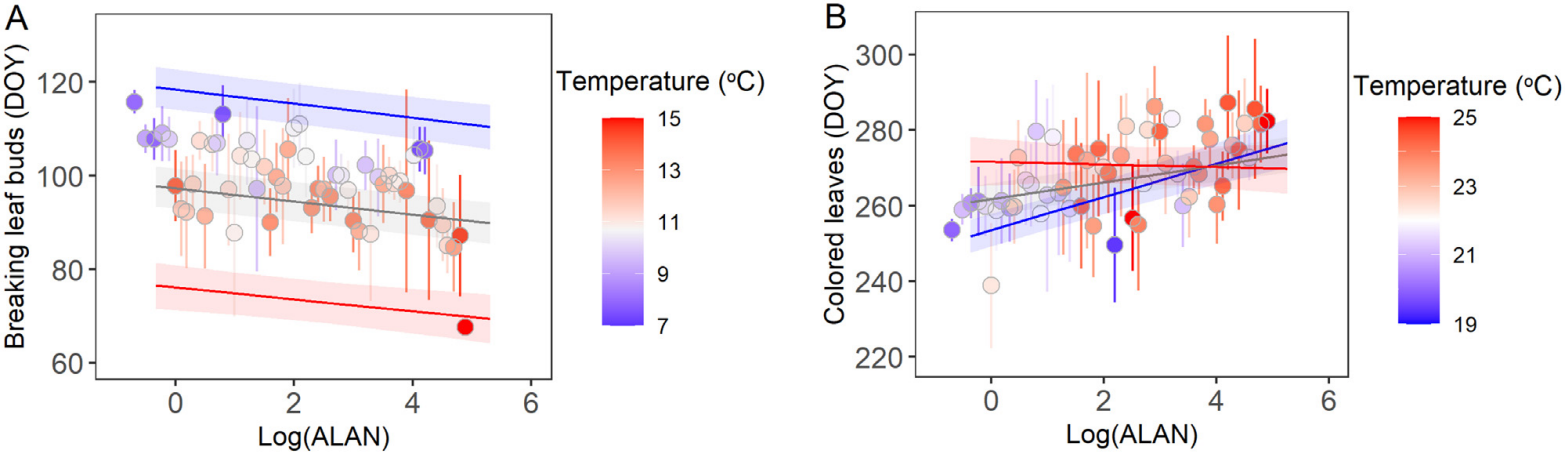
ALAN effects and its interaction with temperature on breaking leaf buds (A) and colored leaves (B). Data are binned for every 0.1 in the logarithm unit of ALAN. The blue, gray, and red lines represent predicted relationships between ALAN and phenology under cold, mild, and warm conditions (i.e. 25%, 50%, and 75% quantiles of temperature) based on linear mixed models presented in Table 1. Shadow areas around each line represent 95% CI.

We found significant interactive effects between ALAN and temperature on colored leaves, but not on breaking leaf buds (*P* < 0.001, Table 1, Fig. 3; Figure S7, Supplementary Material). The responses of breaking leaf buds to increase in ALAN were similar across temperatures, e.g. breaking leaf buds advance 1.52, 1.63, and 1.75 days with an increase in 1 logarithm unit of ALAN (i.e. 172% increase in ALAN) under cold, mild, and warm conditions (i.e. 6.3°C, 12.3°C, and 8.3°C, obtained as 25%, 50%, and 75% quantiles of temperatures), respectively (Fig. 3A). However, the responses of colored leaves to increase in ALAN largely varied across temperatures, i.e. colored leaves delayed by 4.38, 2.25, and −0.35 days with an increase in 1 logarithm unit of ALAN under cold, mild, and warm conditions (i.e. 19.1°C, 22.7°C, and 27.1°C of temperature), respectively (Fig. 3B). The large differences in phenological response indicates considerable spatial divergence in changes of colored leaves under intensifying ALAN.

**Fig. 4.**
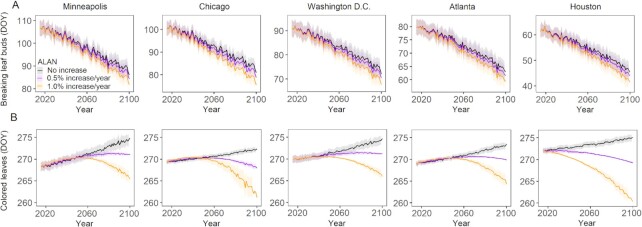
Estimated changes in breaking leaf buds (A) and colored leaves (B) in five cities under future climate warming and intensified ALAN. Lines and shadow area represent the mean and standard deviation, respectively, of estimated phenology using temperatures from 24 climate model simulations for the sixth phase of the CMIP6 under Shared SSP 5 to 8.5 scenario. The results using alternative initial ALAN conditions are shown in Figures S8 and S9 (Supplementary Material).

Finally, we estimated phenological changes under future climate warming and intensified ALAN conditions using linear mixed models (Fig. 4). We selected five representative cities across a wide range of climate gradients for this analysis—Minneapolis, Chicago, Washington DC, Atlanta, and Houston (Figure S1, Supplementary Material). The breaking leaf buds advances 17 to 21 days and the colored leaves delays 4 to 6 days by the year 2100 under warming only scenario in these cities based on temperatures from 24 climate model simulations for CMIP6 Shared Socioeconomic Pathway (SSP) 5 to 8.5 scenario (black lines in Fig. 4). A 0.5%/year and 1%/year increase in ALAN advances breaking leaf buds by 2.3 to 3.0 days and 5.8 to 7.5 days, respectively, which accounts for 11% to 17% and 28% to 42% of climate warming effect (Fig. 4A). The most significant warming effect on breaking leaf buds occurs in Minneapolis (21 days earlier by 2100), while the most considerable ALAN effect occurs in Washington DC (7.5 days under 1%/year increase, Fig. 4A). However, the ALAN effect leads to nonlinear changes in colored leaves in all five cities, i.e. ALAN first accelerates but then slows down and later reverses the warming induced delay in colored leaves by the mid-to-late part of this century (Fig. 4B). Colored leaves advance 3.7 to 4.1 days in these cities by year 2100, under 1%/year increase compared to constant ALAN scenario, which offsets 66% to 83% of climate warming delay effect. We also examined the estimated phenological changes using a different initial ALAN condition (i.e. 65% or 85% quantile of ALAN of each city, see Method) and obtained similar trends (Figures S8 and S9, Supplementary Material), suggesting that the trends of future phenological changes under ALAN are not heavily affected by initial ALAN condition.

## Discussion

The onset of earlier spring phenology and later autumn phenology stages have been documented in cities using remote sensing and ground observations (27–29), which were mainly attributed to urban heat island effect (30–32) in previous studies. ALAN often concurrently occurs with urban heat island effect in cities, thus may affect phenology in similar way of temperature but have not been investigated. In this study, we examined phenology at sites where ALAN and temperature are not highly correlated, and further used a linear mixed model to disentangle ALAN and temperature effects on phenology. Our findings suggest that urban heat island effect on phenology was probably overestimated in previous studies. The phenological changes in cities were the result of combined effects of urban warming and enhanced ALAN, and the latter will likely play an increasingly important role with global urbanization and associated lighting conditions (8). Our findings also reconcile some previously reported results, i.e. satellite observation showed the earlier start of season and later end of season than temperature calculated phenology indicators in highly urbanized areas (29). Such phenological differences between satellite observation and temperature-based estimates are possibly caused by intense ALAN in urban settings.

One possible physiological process through which ALAN affects phenology is by influencing the circadian rhythms of plants (33, 34). The direction, duration, and spectral features of light (including ALAN) are used by plants as sources of information about their location and the day of year, regulating the phase and frequency of their endogenous clock (35–37). ALAN after dusk or before dawn can cause phase shifts in the circadian rhythm, delaying or advancing this cycle. Therefore, the exposure of ALAN may provide misleading cues and cause a false appearance of the lengthened day (38), thus shifting the onset and duration of plant phenology phases. Compared with spring leaf out, autumn leaf senescence has been reported to be more sensitive to photoperiod (5). Such photoperiod sensitivity also changes across latitude; lower latitudes species are likely to be less dependent on day length than higher latitudes species because of the small seasonal variation in light (39). This latitudinal difference in light sensitivity could partly explain the interaction between ALAN and temperature on colored leaves that we reported in this study, i.e. colored leaves show weak response to ALAN under warm climate, but strong response to ALAN under cold climate.

ALAN effects come from both direct illumination (e.g. street lights, vehicle lights, and building light-emitting diode (LED) light) in cities and low-level skyglow (i.e. scattered light in the atmosphere). Therefore, the intrusion of artificial light into ecosystems is already widespread beyond urban areas (e.g. due to major transportation routes cutting through major ecosystems), covering a range of land cover types including natural and managed ecosystems. However, the threshold of ALAN on potential disruption of the circadian clock and how it varies across species are largely unknown. Plants are perceptive to, rather than absolute ALAN intensity at a given wavelength, the ratio of red to far-red light via the phytochrome pathway (14). As the lighting technology has evolved, the color and wavelength of street lights have changed from fluorescent lights (emit almost no red) to high-pressure sodium vapor lights (high red lights), and to current white LED light (emitting at all wavelengths between 400 and 700 nm) (35). Whether these changes in wavelength strengthen or weaken ALAN effect on phenology is still unknown and warrant further investigation.

The effect of ALAN and its interaction with temperature on plant phenology may change under anticipated warming climate conditions. Previous studies suggest that changes in spring phenology are usually more temperature-dependent than daylength-dependent under current climate conditions (1, 38), which is consistent with our findings. However, as climate warming continue to advance spring phenology, phenological response to temperature will decline. This decline is partly due to the photoperiod effect, i.e. the shortened daylength may constrain the warming-induced advancement of spring phenology (40). Moving one step further, our findings suggest that ALAN may supplement the shortened daylength to a certain extent and allow further advance of spring phenology under a warmer climate condition. In contrast to spring phenology, ALAN may shift autumn phenology in a more complex way; ALAN may accelerate the warming-induced delay in autumn phenology in current climate, but slow down or even reserve such delay as the climate warms in the future. This complex change is because ALAN could directly affect autumn phenology, and also influence spring phenology that then affects autumn phenology (41). Leaf senescence was predicted to delay by 1 to 3 weeks under climate warming (42). However, according to findings of this study, this delay may be mitigated or even possibly reversed because of the ALAN effect.

The increasing ALAN may become the driving factor together with the rising temperature that extends the growing season worldwide, causing complex impacts and consequences, potentially both drawbacks and benefits, to the ecosystem and human society. For example, the extended growing season may increase frost risk for plants and cause a mismatch with the timing of other organisms (e.g. pollinators or food sources) and ecosystem functions (35, 43–45). The changes in growing season could also affect the timing and severity of pollen season (46), i.e. earlier spring onset lengthens the pollen season while later onset increases pollen concentrations due to simultaneous blooming. These changes in pollen season will likely increase the risk of pollen allergies for humans (e.g. higher asthma hospitalizations) (47). However, a shift in plant phenology, especially near or in urban areas, could also provide new ecosystem services. A longer growing season may contribute to increased removal of carbon dioxide from the atmosphere (3), the sustained creation of cooler microclimates that can help mitigate the urban heat island effect, shade buildings and lessen their cooling energy consumptions load, slow rainwater runoff, and improve air quality (32, 48). In addition, if agricultural plants are similarly affected by ALAN as deciduous forests, crops may have a higher productivity, particularly in cities where agriculture is colocated with the built environment (e.g. periurban agriculture), but also threatened by urbanization and human activities (e.g. cities in Egypt, China, and India). Whether the shifted phenology of plants in urban areas, as a result of ALAN, is a net gain or loss for ecosystem services and human health is a question that remains to be further studied.

As such, more and better calibrated ALAN data from observational networks and satellite sensors are urgently needed for future studies to further examine the impacts of ALAN on ecosystems. Ecologists and conservation biologists have long neglected to treat ALAN as an essential environmental factor for plants, and to include such effects in designing and developing ecological reserves and corridors. As a result, ecological observation facilities such as The National Ecological Observatory Network (NEON) do not systematically monitor the intensity of ALAN. Supporting infrastructure and protocol are critically needed to add ALAN monitoring in existing ecological data collection protocols, especially for the sites near cities or transport networks/corridors. In the future, developing an independent in situ ALAN data collection network, with a focus on urban ecosystems, should also be considered. These efforts would provide ALAN observation with more spectral information at higher temporal resolution (e.g. hourly) across ecosystems. At the same time, better calibrated ALAN data from satellite sensors should also be included routinely as a part of new Earth Observing systems and environmental/ecological monitoring protocols. DNB data that account for the spectral variation in ALAN data products at higher temporal resolution will play a critical role in understanding the impact of changes in lighting conditions, the wavelengths spectrum, seasons, and light sources. For example, space-based observations from geostationary orbit or ideally from L-2 libration orbit that covers Earth at night from pole-to-pole will address the temporal resolution needed for diurnal effects of ALAN at regional to global levels.

With better characterizing and incorporating feedbacks between ALAN and plant phenology into Earth system models, changes in land surface processes with consideration to human activities could be better assessed and predicted. Besides affected by temperature and ALAN, phenology may also be influenced by other factors such as the expansion of roads, soil erosion, and chemical compounds and pollutants such as ozone in cities (49, 50). Additionally, the stronger urban heat island intensity at night compared to the daytime could lead to a different diurnal pattern of temperature, which might affect plant phenology in complex ways. Temperature datasets at finer temporal resolutions are needed to understand the effect of diurnal temperature variations on phenology. Moreover, heat stress, more frequent and severe in the warm region (51), can influence water conditions (e.g. seasonal rainfall, soil moisture availability, and relative humidity) that is critical for phenology of semiarid, deserts, and subtropical regions (e.g. in southern and western United States) (52). The challenge of disentangling the confounding and combined effects of these facets of urbanization in tandem with ALAN will continue to be a major area of research (53), for the preservation and management of the embedded and surrounding urban ecosystem, especially under anticipated and continued urbanization and the trend in increasing white (i.e. LED-based) ALAN.

In summary, our results provide direct observational evidence that ALAN, an underappreciated environmental factor as a result of urbanization, is contributing to observed changes in plant phenology in the conterminous United States. Our findings suggest a need to incorporate anthropogenic environmental changes such as ALAN into ecological observatories and model-based syntheses, which is currently lacking, for more reliable assessment and representation of biosphere–atmosphere feedbacks in the human–natural systems. The continuously increasing ALAN may have significant and far-reaching consequences in disrupting key ecosystem functions, ecological processes, and ecosystem services with a significant impact on human health and well-being (46, 54). Although previous studies have advanced our understanding of ALAN effects on the behavior and physiology of animals (11, 20), its ecological impacts and their consequences for plants are far less studied (55). Our study identified an opportunity for greater attention and focus on this area as an emerging topic in global change ecology, i.e. the effect of ALAN on ecosystems dynamics and its interaction with climate warming. Such research will have urgent and significant implications for biodiversity conservation in urban, suburban, and by extension in rural and natural ecosystems. This emerging topic requires an interdisciplinary approach for ecologists to work with city planners, engineers, and architects to implement policies and practices that consider the influence of ALAN on plants, and to sustain ecosystem functions and services for humans and other species. Fortunately, some improved techniques to substantially reduce light pollution (56) and ongoing efforts such as Light-Pollution Abatement to reduce unnecessary glare, uplight, and light trespass by advising governments, local municipalities together with action oriented initiative by businesses and citizens (57) are already available and demonstrate some desirable impact. Such efforts will be a major contribution to ensuring “healthy planet, healthy people” now and in the future.

## Materials and Methods

### Phenology dataset

We obtained and used phenological observations from the Phenology Observation Portal of USA National Phenology Network (USA-NPN, https://www.usanpn.org/data/observational). USA-NPN is a nationwide phenological network in the United States, with more than 9 million records for more than 700 plant species, including phenology data collected via *Nature*’s Notebook phenology program and additional integrated datasets (58). The data we used in this study is site phenometrics data, which includes estimates of the overall onset and end of phenophase activity for plant species at a given site for a user-defined period. These data provide the first and last occurrences of a given phenophase for a given species, beginning with the date of the first observed “yes” phenophase status record and ending with the date of the last observed “yes” record of the user-defined period. Because there are multiple individual observations that are obtained for each species at a given site, we used the mean of the first “yes” records for each individual plant species at that site (Mean_First_Yes_DOY).

We focused on the plant functional type of deciduous broadleaf, which has the most recorded observations, and two phenological stages, i.e. breaking leaf buds in spring and colored leaves in autumn, during 2011 to 2016. The phenological stage of breaking leaf buds is defined as one or more breaking leaf buds are visible on the plant (58). A leaf bud is considered “breaking” once a green leaf tip is visible at the end of the bud, but before the first leaf from the bud has unfolded to expose the leaf stalk or leaf base. The phenological stage of colored leaves is defined as one or more leaves (including any that have recently fallen from the plant) have turned to their late-season colors (58). This definition does not include fully dried or dead leaves that remain on the plant. To reduce the uncertainty of observation, we removed the records of breaking leaf buds after the day-of-year 183 and the records of colored leaves before the day-of-year 182. To constrain the species effect, we only focus on the species with more than 80 records of observation of breaking leaf buds and more than 40 records of observation of colored leaves, respectively. In total, 2,952 site-year records of 17 species from 907 sites for breaking leaf buds and 2,148 site-year records of 23 species from 628 sites for colored leaves were used in this study (Fig. 1; Table S1, Supplementary Material).

### ALAN dataset

We used the recently released nighttime lights product in the Black Marble suite, i.e. VIIRS/NPP Lunar BRDF-Adjusted Nighttime Lights Yearly L3 Global 15 arc-second Linear Lat Lon Grid (VNP46A4, https://ladsweb.modaps.eosdis.nasa.gov/missions-and-measurements/products/VNP46A4/) (23). VIIRS DNB provides high-resolution and better calibrated low light imaging data at night in spectral bands (0.5 to 0.9 μm centered at 0.7 μm) covering emissions generated by electric lights. Such ultrasensitivity in lowlight conditions enable generating high-quality ALAN products that manifest substantial improvements compared to the previously widely used Defense Meteorological Satellite Program/Operational Linescan System's (DMSP/OLS) ALAN products. NASA's Black Marble product suite (VNP46) is a new suite of standard products representing the current state-of-the-art ALAN observations developed from the VIIRS DNB time series record. This product provides yearly composites at 15 arc-second spatial resolution generated from daily atmospherically and lunar-BRDF-corrected ALAN radiance to remove the influence of extraneous artifacts and biases. The retrieval algorithm utilizes all high-quality, cloud-free, atmospheric-, terrain-, vegetation-, snow-, lunar-, and stray light-corrected radiances to estimate daily ALAN and other intrinsic surface optical properties. In addition, the algorithm estimates the actual moonlight, aerosol, and surface albedo contribution through analytical BRDF model inversion, which has proven effective in removing biases introduced by extraneous sources of nighttime lights emissions. This algorithm also mitigates errors stemming from the poor-quality top of the atmosphere retrievals, especially across regions with heavy aerosol loadings and at Moon/sensor geometries yielding stronger forward scatter contributions, by using vector radiative transfer modeling of the coupled atmosphere surface system to compensate for aerosols, water vapor, and ozone impacts on the ALAN radiances. The DNB includes on-board calibration and is reported in radiance units (nW/cm^2^/sr). We used ALAN annual composites in 2012 to 2016 period. Due to the unavailability of ALAN data of the year 2011, we used the annual composites of 2012 for 2011 in this study. We extracted the ALAN values for each phenological site each year based on the latitude and longitude of the site.

### Temperature datasets

The temperature data from 2011 to 2016 were obtained from the TopoWx dataset (http://www.scrimhub.org/resources/topowx/). TopoWx is a gridded dataset of daily minimum $({T_{min}}$) and maximum (${T_{max}}$) air temperature at 800 m by 800 m resolution for the conterminous United States. It was interpolated from station observations, digital elevation model variables, atmospheric reanalysis data, and MODIS land skin temperature (59). TopoWx has been proved to adequately capture locally relevant topo-climate and biophysical spatial patterns (e.g. characteristics of urban heat island effect) (60), compared to other temperature gridded datasets. Therefore, using TopoWx enabled us to better represent the urban heat island effect and disentangle ALAN effect from urban heat island effect in our study sites. It is worth noting the land surface temperature dataset used in developing TopoWx has a newer version, but TopoWx used in this study did not reflect these new improvements (e.g. improved day/night algorithm by adjusting weights in desert regions) (59, 61, 62). The daily average temperature was calculated as the mean of daily ${T_{max}}$ and ${T_{min}}$. We calculated average spring (March 1st to May 31st) temperatures and average summer (June 1st to August 31st) temperatures from daily air temperature for each phenological site and each year to investigate the temperature effect on phenology. We also calculated alternative mean temperatures, i.e. February 1st to May 31st, January 1st to May 31st, and December 1st to May 31st for breaking leaf buds, and June 1st to September 31st, June 1st to October 31st, and June 1st to November 31st for colored leaves from daily air temperature to test the robustness of our results.

To extend our findings for assessing future phenology changes, we estimated phenology using the projected monthly air temperature for the CMIP6 model simulations under Shared SSP 5 to 8.5. The CMIP6 data underpins the Intergovernmental Panel on Climate Change 6th Assessment Report, providing estimates of future climate change and related uncertainties. We downloaded the global temperature projections from 24 climate models in the Climate Data Store subset of CMIP6 data (Table S4, Supplementary Material), which has been through a quality control procedure and ensures a high standard of dependability of the data from (https://cds.climate.copernicus.eu/cdsapp#!/dataset/projections-cmip6?tab = overview). Although the CMIP6 projections are not downscaled, the multiple models we used provide consistent projections with the ranges and uncertainties of future projection. The SSP scenarios provide different pathways of potential future climate conditions for the 2006 to 2100 period. We selected the SSP 5 to 8.5 in order to show the maximum temperature effect on phenology. We extracted five grids from the global gridded CMIP6 temperature datasets using the latitude and longitude of the center of the five cities in this study. For each city, we calculated the mean spring (March 1st to May 31st) temperature and mean summer (June 1st to August 31st) temperature from monthly temperature during 2015 to 2100 period and used them to estimate the breaking leaf buds and colored leaves stages, using their linear mixed models, respectively. We conducted the analysis using temperature projections from 24 climate models, respectively, and showed the results as the mean and standard deviation from 24 models. It is worth noting that temperature projection from CMIP6 does not consider urban warming at a local-scale (63), which could be intensified at certain regions.

### ALAN effect quantification

Because most sites have a low intensity of ALAN (< 1 nW/cm^2^/sr) and few sites have extreme high intensity of ALAN, we used log10-transformed ALAN to achieve a normal distribution of ALAN that meet the assumption of statistical analysis (Figure S1, Supplementary Material). Although ALAN is often considered to be highly correlated with temperature in cities due to urbanization, we found that the log10-transformed ALAN and temperature were not highly correlated across our study sites in the conterminous United States (Figure S10, Supplementary Material; *Pearson's r* = 0.19 for breaking leaf buds and *Pearson's r* = 0.32 for colored leaves). This noncorrelation is because the variation of temperature is mainly from latitude and topography at a national scale, while the variation of ALAN is not.

To test whether ALAN induces phenological changes, we first binned the phenological data at every 1°C temperature increment and calculated the phenological difference for sites with ALAN (75% quantile of ALAN) and without ALAN (radiance = 0 nW/cm^2^/sr), for each temperature increment. We conducted a sensitivity test on the ALAN threshold that defines ALAN site and eventually chose 75% quantile of ALAN as the threshold to fully show the shift in phenology caused by ALAN while ensure enough sample size to guarantee a statistically sound comparison. We used the Wilcoxon signed-rank test to test whether the phenological differences between ALAN and non-ALAN groups are significant. Wilcoxon test is the nonparametric of the dependent samples t test, which often requires metric and normally distributed data. Because Wilcoxon test is a nonparametric test, it does not require a particular probability distribution of the dependent variable in the analysis. Therefore, Wilcoxon test is often used when the assumptions of dependent samples t test are not met. If not specified using Wilcoxon test, statistical significance was determined based on a two-tailed Student's t test. To remove the large uncertainties of the small size group, we only focused on the temperature increment with more than 8 site-year observations.

The relevant periods for breaking leaf buds and colored leaves are typically 1 to 3 months prior to the phenological events (64). However, because it is impossible to select one period that fits all species and locations, we used spring (March 1st to May 31st ) mean temperature and summer (June 1st to August 31st) mean temperatures to represent temperature effect for breaking leaf buds and colored leaves, respectively, based on previous empirical and modeling studies (65, 66). This approach represents a simple and reasonable way to describe the temperature condition across largely spatiotemporal scales and it has been widely used (64, 67). To test the robustness of the results and eliminate the effects of choosing different temperature periods, we further examined ALAN-induced phenological changes using alternative temperatures, i.e. mean temperature during February 1st to May 31st, January 1st to May 31st, and December 1st to May 31st for breaking leaf buds, and mean temperature during June 1st to September 31st, June 1st to October 31st , and June 1st to November 31st for colored leaves.

To investigate ALAN effect at species level, we compared breaking leaf buds of the top 10 species and colored leaves of the top eight species, which have the most observations, with and without ALAN. To constrain temperature effect and only quantify ALAN-induced phenological changes, we selected phenology observations within a narrow temperature range, i.e. 50% quantile ± 0.5 × SD of all site-year temperatures for breaking leaf buds and 25% quantile ± 0.8 × SD for colored leaves. The reason we used a wider window for colored leaves compared to breaking leaf buds is to make sure we had enough sample for both ALAN and non-ALAN groups. Then we compared the density distribution of phenology with and without ALAN, and tested whether the phenological differences between ALAN and non-ALAN sites were related to the phenology itself across species. To examine whether ALAN-induced phenological changes is related to the phenology itself, we compared the magnitude of phenological changes to the mean phenology between ALAN and non-ALAN sites for each species within the above temperature range. All analyses were conducted in R version 3.6.1 (68).

### Bootstrapping

To remove the influence of extreme climate and outlier phenological observation for each temperature group when quantifying the ALAN effect, we employed a nonparametric bootstrapping method for breaking leaf buds and colored leaves, respectively. We generated 5,000 subsamples of phenology in each temperature group for ALAN and non-ALAN sites, respectively. Each subsample was generated with replacement so that the data in any year or site can be sampled multiple times or not sampled at all. The computed mean phenology provided an estimate of the sampling distribution of phenology so that extreme climate cases or outlier phenological observation could be excluded from the results.

### Linear mixed model

We used the linear mixed model to quantitatively explain the variations in phenology using factors including temperature, log-ALAN, species, and year. Linear mixed models have been shown to be powerful tools for analyzing complex ecological data with repeated or clustered observations (69). As an extension of simple linear models, linear mixed models allow both fixed and random effects as predictor variables, and it is particularly used when there are nonindependence observational units that are hierarchical in the data (69). A fixed-effect is a parameter that does not vary, while random effects are parameters that are themselves random variables. Here, we treated temperature, log-ALAN, and the interaction between them as fixed terms. Our phenological dataset has replicated observations of deciduous woody plants species across sites and over the year. There is a high likelihood that measurements within the same unit (i.e. species or year in this case) might be more similar than measurements from different units. Explicit modeling of these random effect structures will aid correct inference about the fixed effects. Therefore, we used the categorical variables of species and years as the random component for the intercept. We conducted normalization (every observation minus the mean) for the variables of temperature, log-ALAN, and phenological dates before applying linear mixed model. We did not find any trend in the model residual. During the interpretation of the linear mixed model, we connected the changes in log transformations of ALAN to changes in actual ALAN. The estimated coefficient represents that a one-unit increase in log (ALAN) or 172% increase in ALAN will produce an expected increase in Y (i.e. phenology).

### Phenological prediction

We estimated future phenological changes based on the linear mixed models for breaking leaf buds and colored leaves, respectively. We selected Minneapolis, Chicago, Washington DC, Atlanta, and Houston as five representative cities of a wide range of climate conditions to examine phenological change under future climate and ALAN scenarios. These cities are highly urbanized and show intense ALAN (Figure S1, Supplementary Material). The mean spring temperatures are 7.3, 8.7, 13.0, 16.0, and 20.5°C for Minneapolis, Chicago, Washington DC, Atlanta, and Houston during 2011 to 2016 (Table S5, Supplementary Material), respectively, very close to the 25%, 50%, 75% quantile of temperatures in Fig. 3. The spring and summer temperatures in these five cities are projected to increase by 4.94 to 6.11°C and 5.42 to 7.61°C (mean of 24 climate models in CMIP6) from 2015 to 2100 (Figure S11, Supplementary Material), respectively.

To examine the effect of ALAN intensity, we used three ALAN scenarios (i.e. no increase, 0.5%/year increase, and 1%/year increase). The no increase ALAN scenario serves as a benchmark to show the warming effect only. The two increasing ALAN scenarios are used to quantify the effect of ALAN intensity on the magnitude of phenological changes. We designed two increasing scenarios at the rates of 0.5%/year and 1%/year based on the mean global ALAN increasing rate 1.8% during 2012 to 2016 (8) and the general trend of ALAN, i.e. rapid increase in early urbanization, then slows down and reaches a plateau at highly urbanized stage. It is worth noting that these two scenarios are simplified scenarios with constant increasing rate, and it may not fully represent the complex ALAN dynamics in real world. We calculated annual ALAN from 2015 to 2100 based on the constant increasing rate and an initial ALAN value (i.e. 75% quantile of all ALAN grids of each city in 2016, Table S5 and Figure S12, Supplementary Material). We also conducted two sensitivity tests using initial ALAN values of 65% and 85% quantiles of ALAN. Assuming a constant phenological response to temperature and ALAN, we then estimated the breaking leaf buds and colored leaves during 2015 to 2100 based on their linear mixed models using temperature and ALAN as input for the fixed effects and using the species *Acer rubrum*, and year 2016 for the random effects.

## Supplementary Material

pgac046_Supplemental_File

## Data Availability

All data needed to evaluate the conclusions in the paper are present in the paper and/or the Supplementary Materials.
