# Supplementary material for: Artificial light at night: an underappreciated effect on phenology of deciduous woody plants
Source: PNAS Nexus. 2022 Apr 18;1(2):pgac046. doi: 10.1093/pnasnexus/pgac046 (PMC9802268; doi:10.1093/pnasnexus/pgac046)
Supplement: pgac046_Supplemental_File [file pgac046_supplemental_file.docx]

Supplementary Materials for

**Artificial light at night: an under-appreciated effect on phenology of deciduous woody plants**

Lin Meng^1^, Yuyu Zhou^1^*, Miguel O. Román^2^, Eleanor C. Stokes^2,3^, Zhuosen Wang^3,4^, Ghassem R. Asrar^2^, Jiafu Mao^5^, Andrew D. Richardson^6,7^, Lianhong Gu^5^, Yiming Wang^1^

*Corresponding author. Email: [yuyuzhou@iastate.edu](mailto:yuyuzhou@iastate.edu)

**This PDF file includes:**

Figs. S1 to S12

Table S1-S5


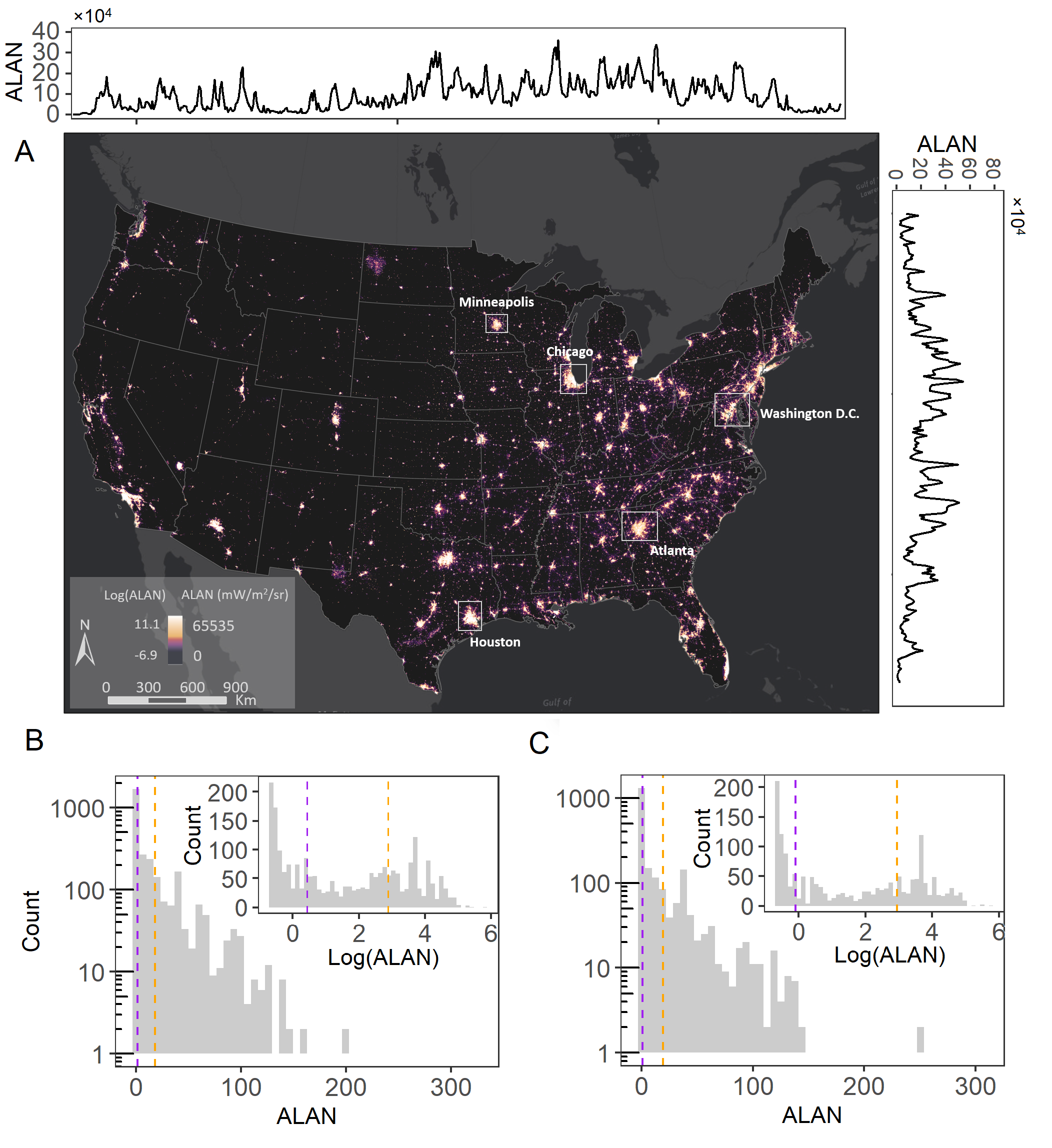


**Fig. S1 Artificial light at night (ALAN) image (A) and the histogram of ALAN at phenological sites of breaking leaf buds (B) and colored leaves (C) in the conterminous United States in 2016.** The latitudinal and longitudinal sum of ALAN as well as five study cities are shown in (A). B-C: The inserted subfigures show the histograms of pixels with ALAN>0 and use log10-transformed ALAN on *x-axis*. The orange and purple vertical lines represent the 75% and 50% quantiles of ALAN, respectively.


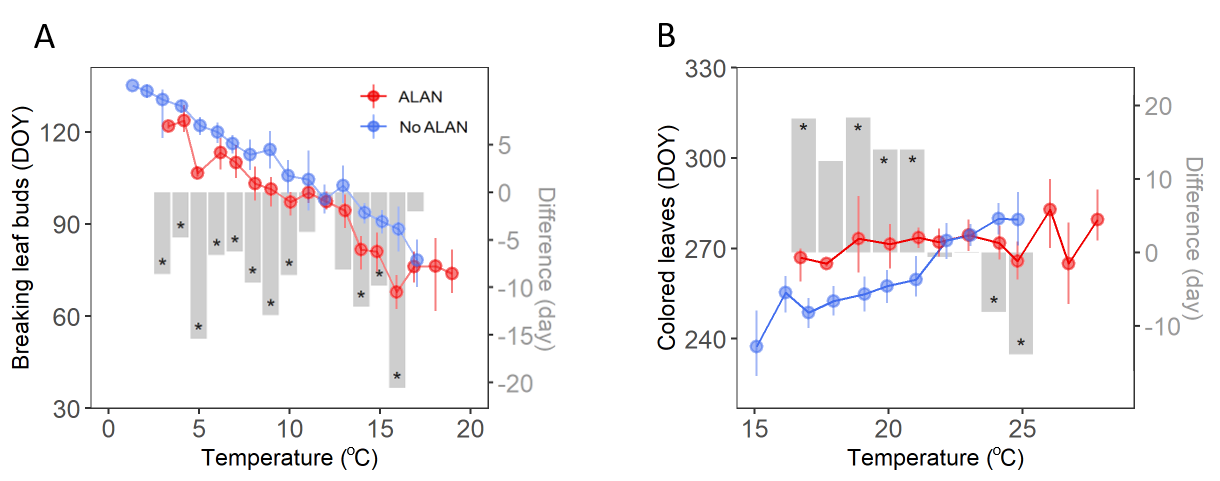


**Fig. S2 Differences in breaking leaf buds (A) and colored leaves (B) for sites with artificial light at night (ALAN) versus those without ALAN across temperatures.** Same as Fig. 2 but used bootstrapping method. Points and error bars represent the mean and 95% confidence interval of phenology (day of the year) for each 1 ºC temperature increment obtained from 5000 resampling using bootstrap. Statistical significance level at *P* < 0.05 is shown as an asterisk.


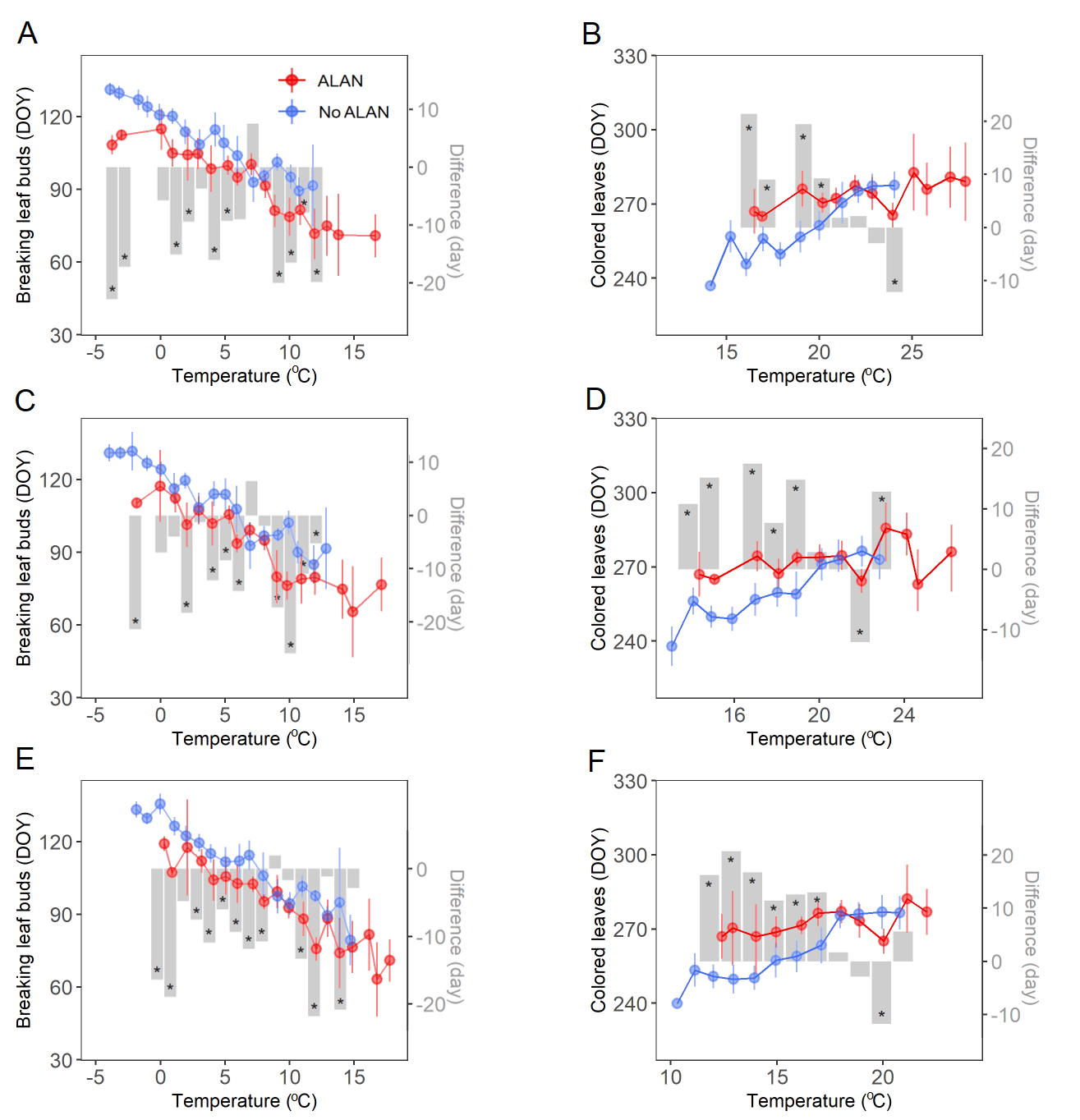


**Fig. S3** **Differences in breaking leaf buds and colored leaves at artificial light at night (ALAN) versus no ALAN sites under similar temperature conditions.** Same as Fig. 2 but used alternative temperatures, i.e., mean temperature during February 1^st^ to May 31^st^ (A), January 1^st^ to May 31^st^ (C), December 1^st^ to May 31^st^ (E) for breaking leaf buds, and mean temperature during June 1^st^ to September 31^st^ (B), June 1^st^ to October 31^st^ (D), June 1^st^ to November 31^st^ (F) for colored leaves. The statistical significance level at *P* < 0.05 is shown as an asterisk.


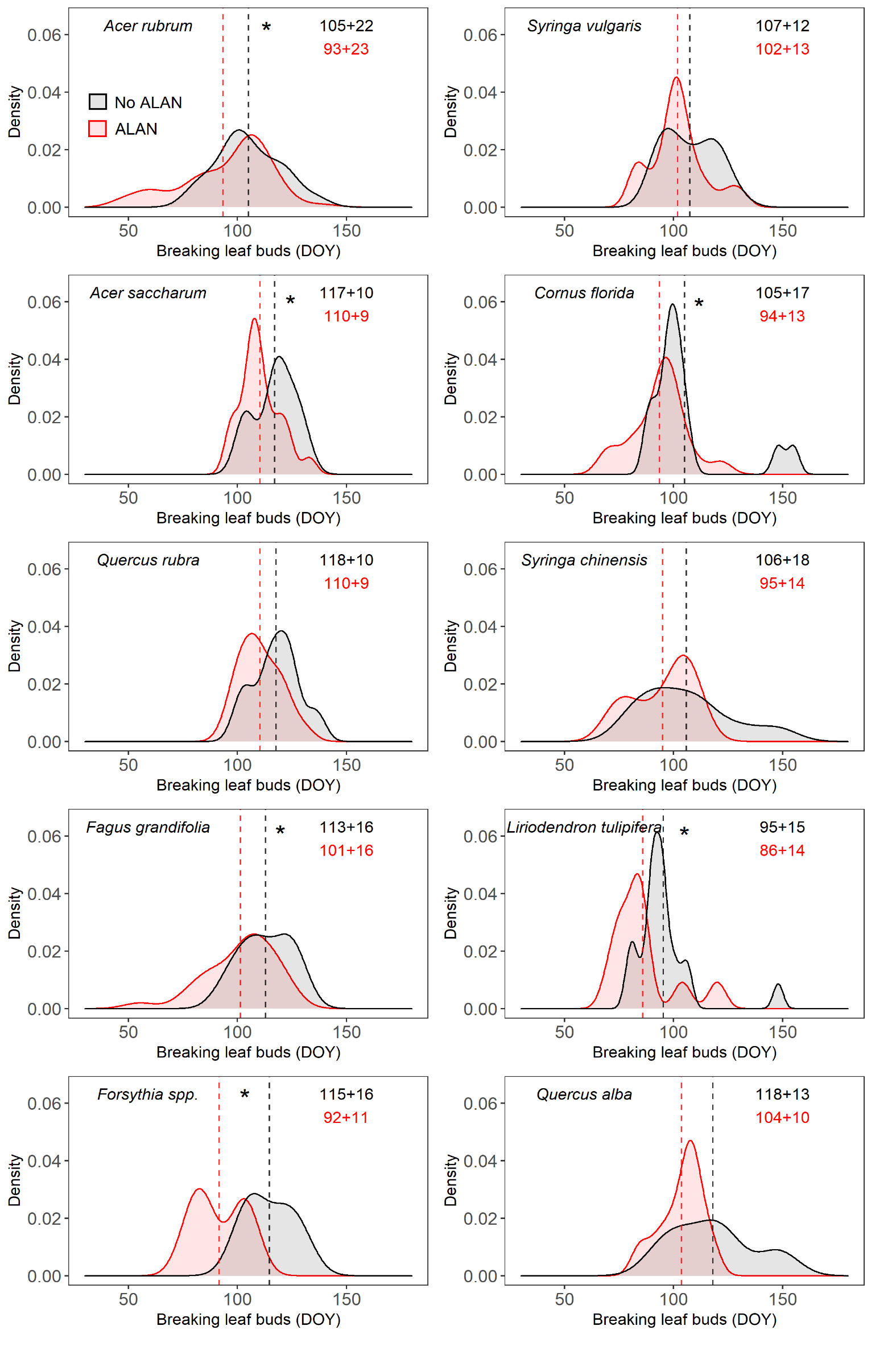


**Fig. S4 Density of** **breaking leaf buds for each species at ALAN versus no ALAN sites under similar temperature conditions.** The phenology observations are selected within a narrow temperature range, i.e., 50% quantile ± 0.5 × standard deviation of all site-year temperatures. The dotted lines represent the mean of breaking leaf buds. The mean ± standard deviation of breaking leaf buds at ALAN and no ALAN sites, and the significance level of their difference from the two-tailed Student’s *t*-test are shown (*, *P* < 0.05). The top ten species with the most observations are shown here. The ALAN sites are those with higher than 75% quantile of ALAN among all sites (i.e., 19.1 nW/cm^2^/sr).


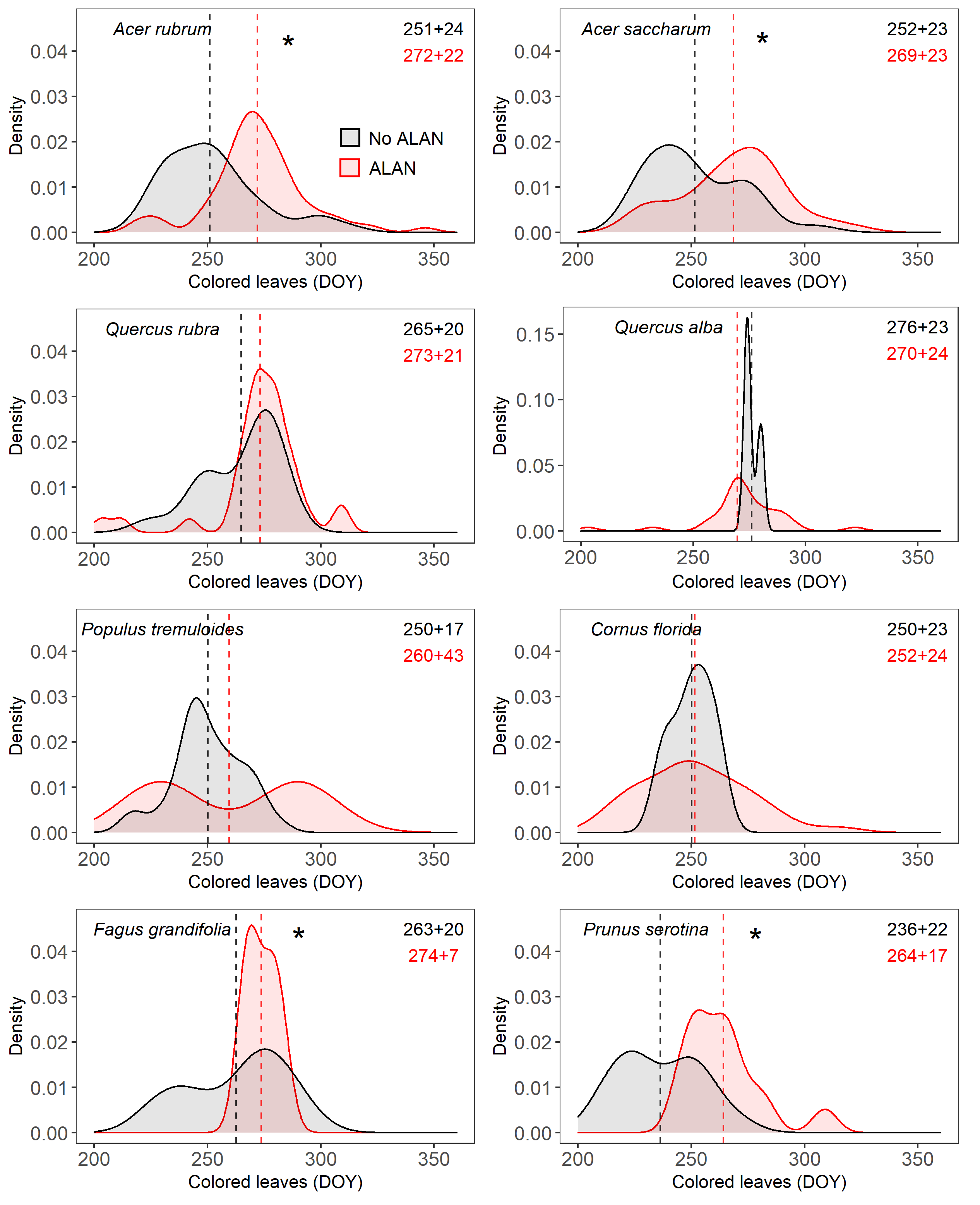


**Fig. S5 Density of colored leaves for each species at ALAN versus no ALAN sites under similar temperature conditions.** The phenology observations are selected within a narrow temperature range, i.e., 25% quantile ± 0.8 × standard deviation of all site-year temperatures. The dotted lines represent the mean of colored leaves. The mean ± standard deviation of colored leaves at ALAN and no ALAN sites, and the significance level of their difference from two-tailed Student’s *t*-test are shown (*, *P* < 0.05). The top eight species with the most observations are shown here. The ALAN sites are those with higher than 75% quantile of ALAN among all sites (i.e., 19.1 nW/cm^2^/sr).


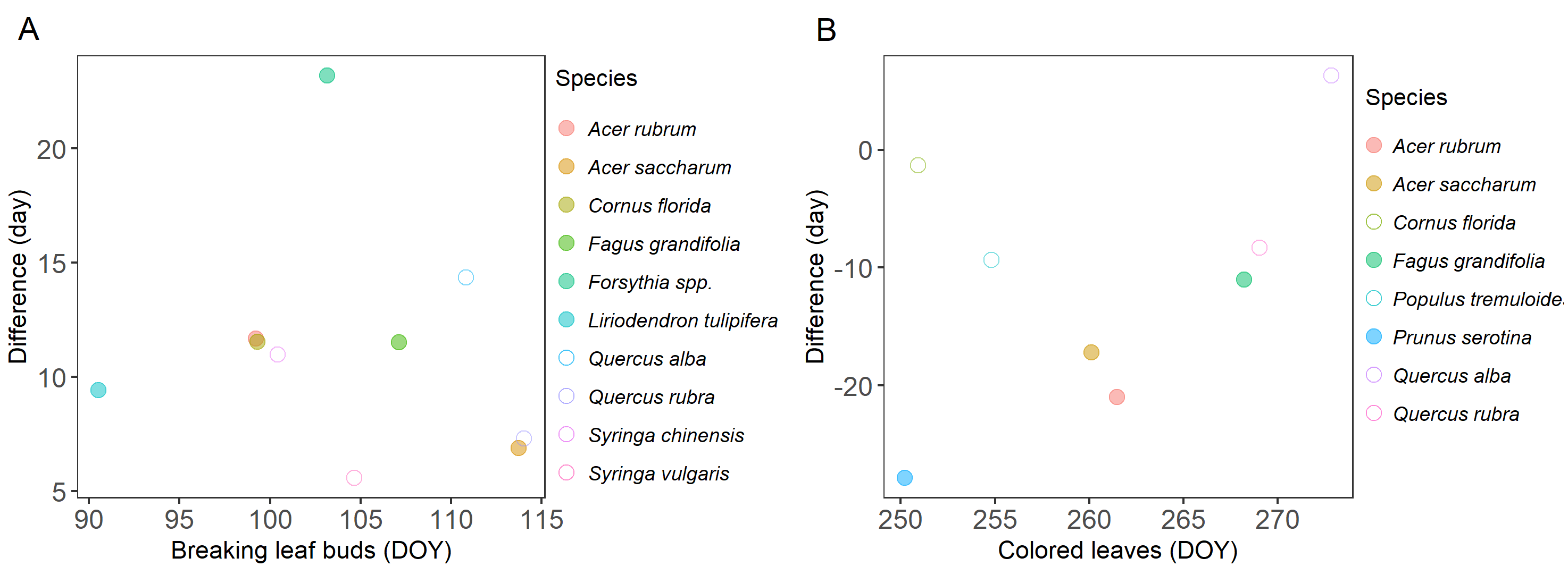


**Fig. S6 Relationship between ALAN-****driven phenological difference and phenology for breaking leaf buds (A) and colored leaves (B) for each species.** The ALAN-driven phenological difference was obtained from Fig. S4-S5 by subtracting phenology at sites with ALAN from sites without ALAN. The breaking leaf buds (A) and colored leaves (B) in the *x*-axis were the mean of sites with ALAN and without ALAN from Fig. S4-S5. The phenology observations are selected within a narrow temperature range, i.e., 50% quantile ± 0.5 × standard deviation of all site-year temperatures for breaking leaf buds (A) and 25% quantile ± 0.8 × standard deviation for colored leaves (B). Solid points indicate a significant (*P* < 0.05) difference in phenology at sites with and without ALAN.


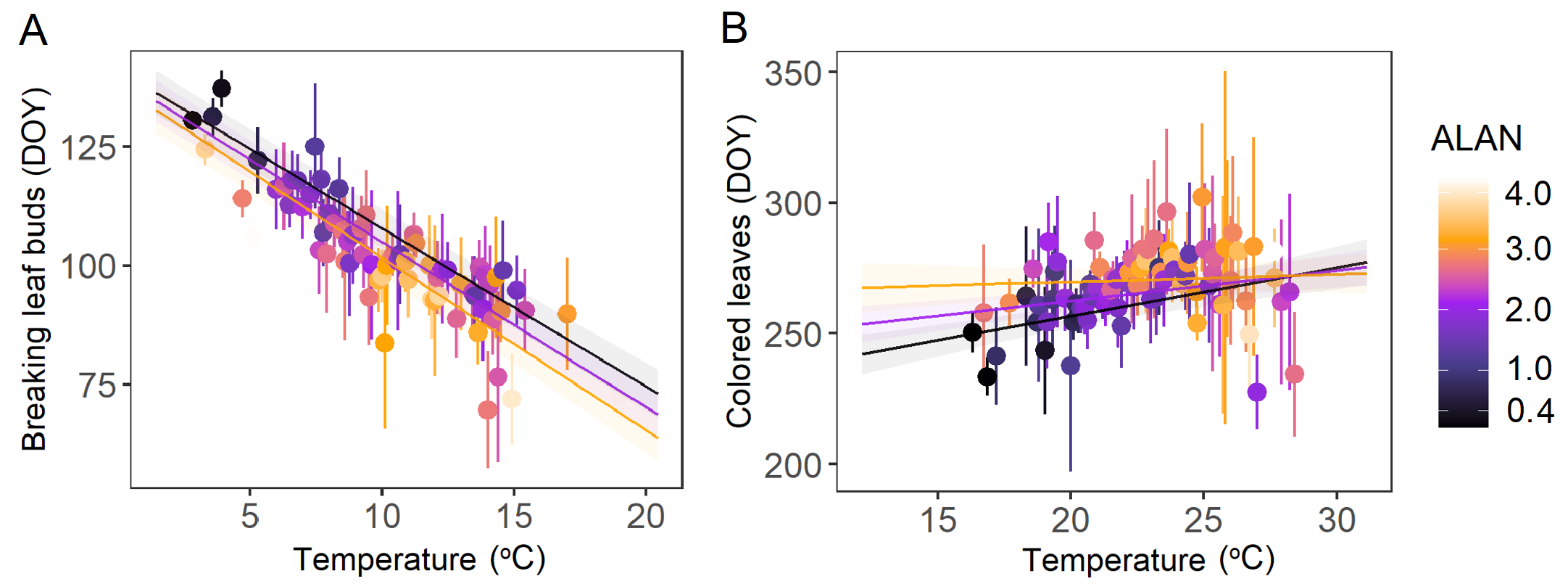


**Fig. S7 Temperature effects and its interaction with ALAN on breaking leaf buds (A) and colored leaves (B).** Data is binned at every 1 ºC in temperature. The black, purple, and yellow lines represent predicted relationships between temperature and phenology under three ALAN conditions (i.e., 25%, 50%, and 75% quantiles of log(ALAN)) based on linear mixed models in Table 1. Shadow areas represent 95% confidence interval.


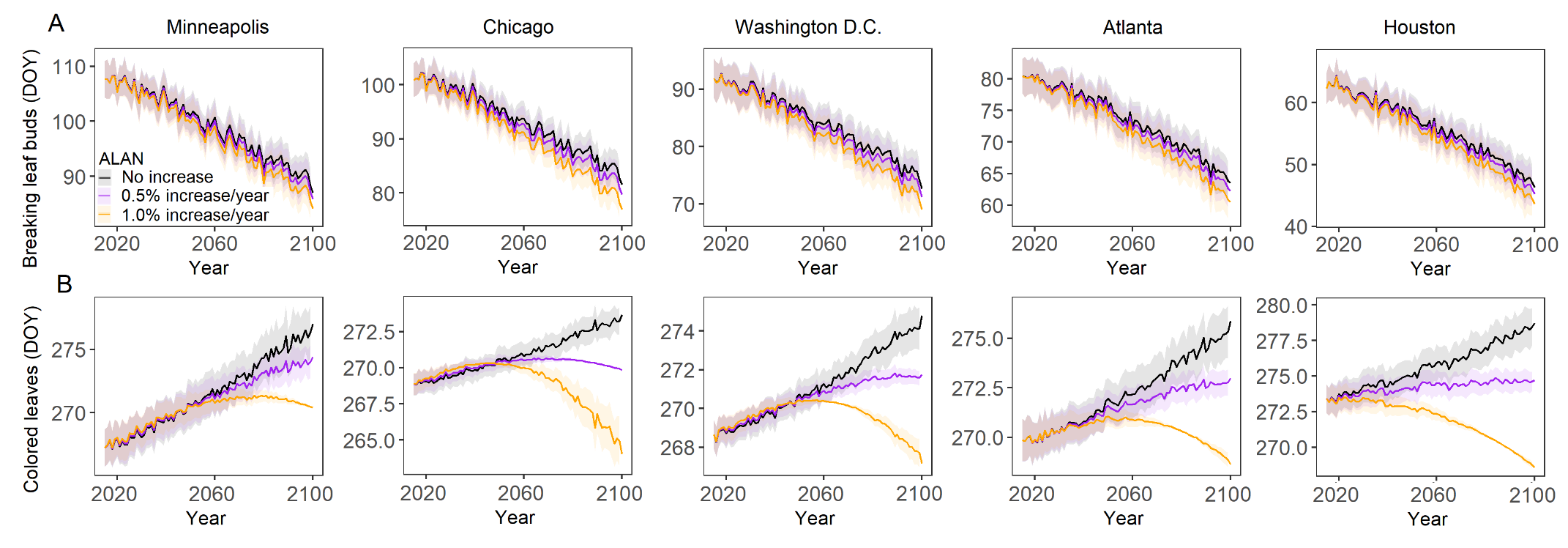


**Fig. S8 Future changes in breaking leaf buds (A) and colored leaves (B) in five study cities under three ALAN scenarios and CMIP6 SSP 5-8.5.** Same as Fig. 4 but used an ALAN starting value of 65% quantile of ALAN for each city.
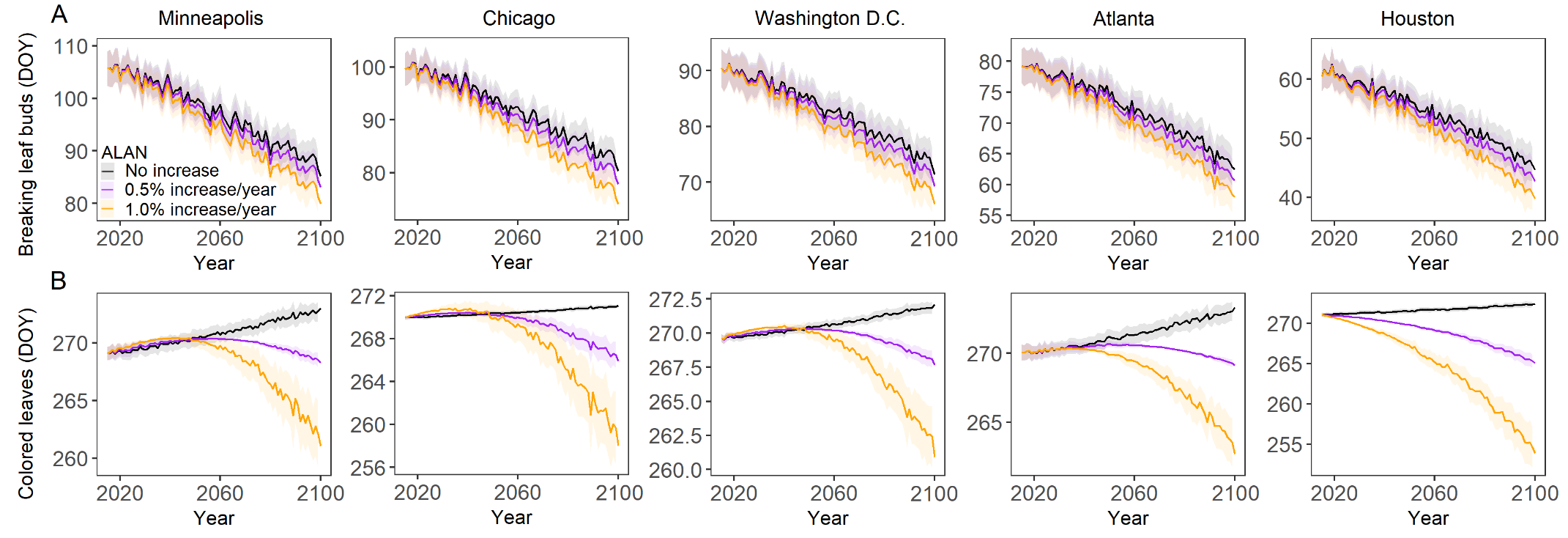


**Fig. S9 Future changes in breaking leaf buds (A) and colored leaves (B) in five study cities under three ALAN scenarios and CMIP6 SSP 5-8.5.** Same as Fig. 4 but used an ALAN starting value of 85% quantile of ALAN for each city.


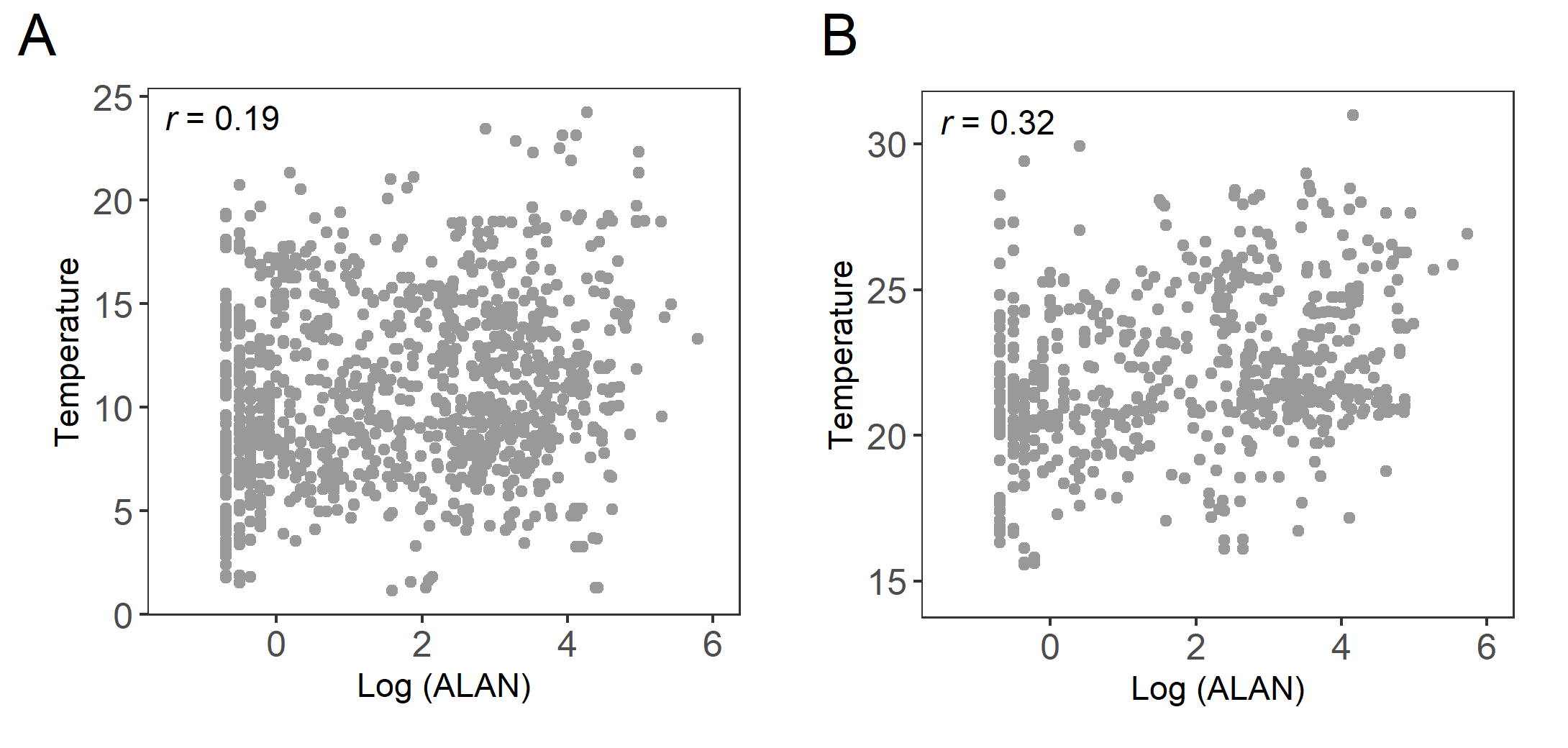


**Fig. S10 The relationship between temperature and ALAN in phenological sites for breaking leaf buds (A) and colored leaves (B).** Pearson’s correlation coefficient *r* is shown in the figure.


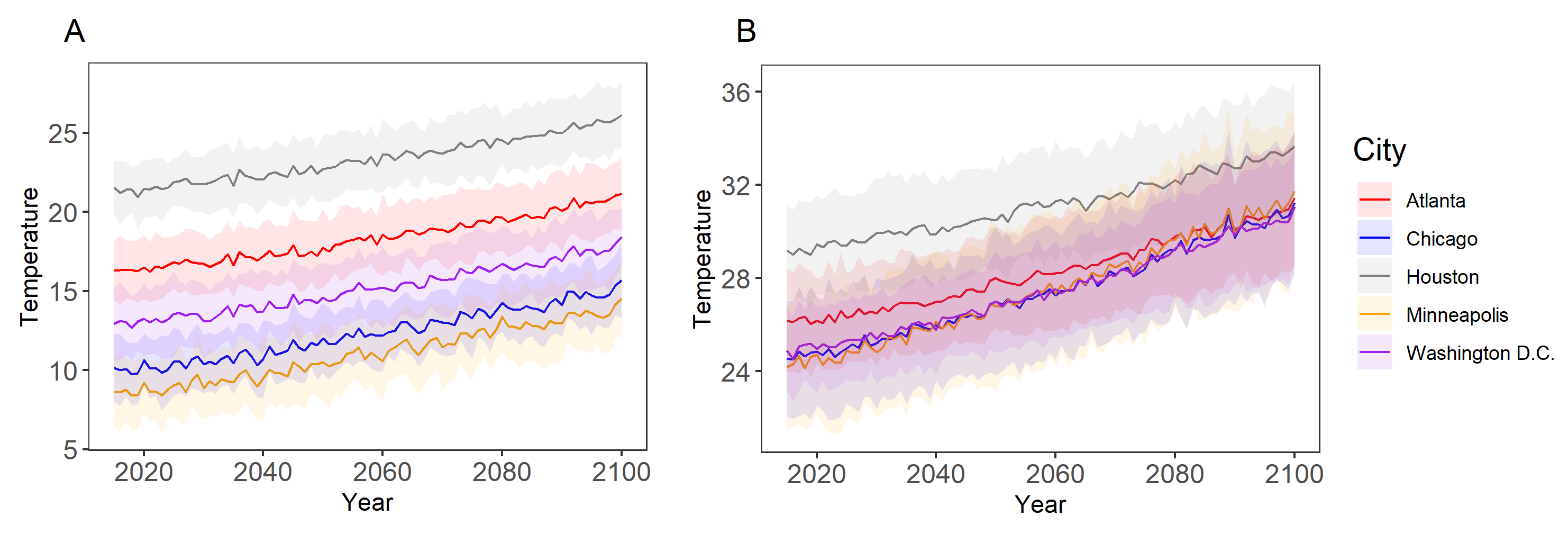


**Fig. S11 Projection of mean spring temperature (A) and summer temperature (B) from 24 climate models under CMIP6 SSP5-8.5 scenarios for the five cities.** Lines and shadow areas are the mean and 95% confidence interval of temperature projection from 24 models, respectively.


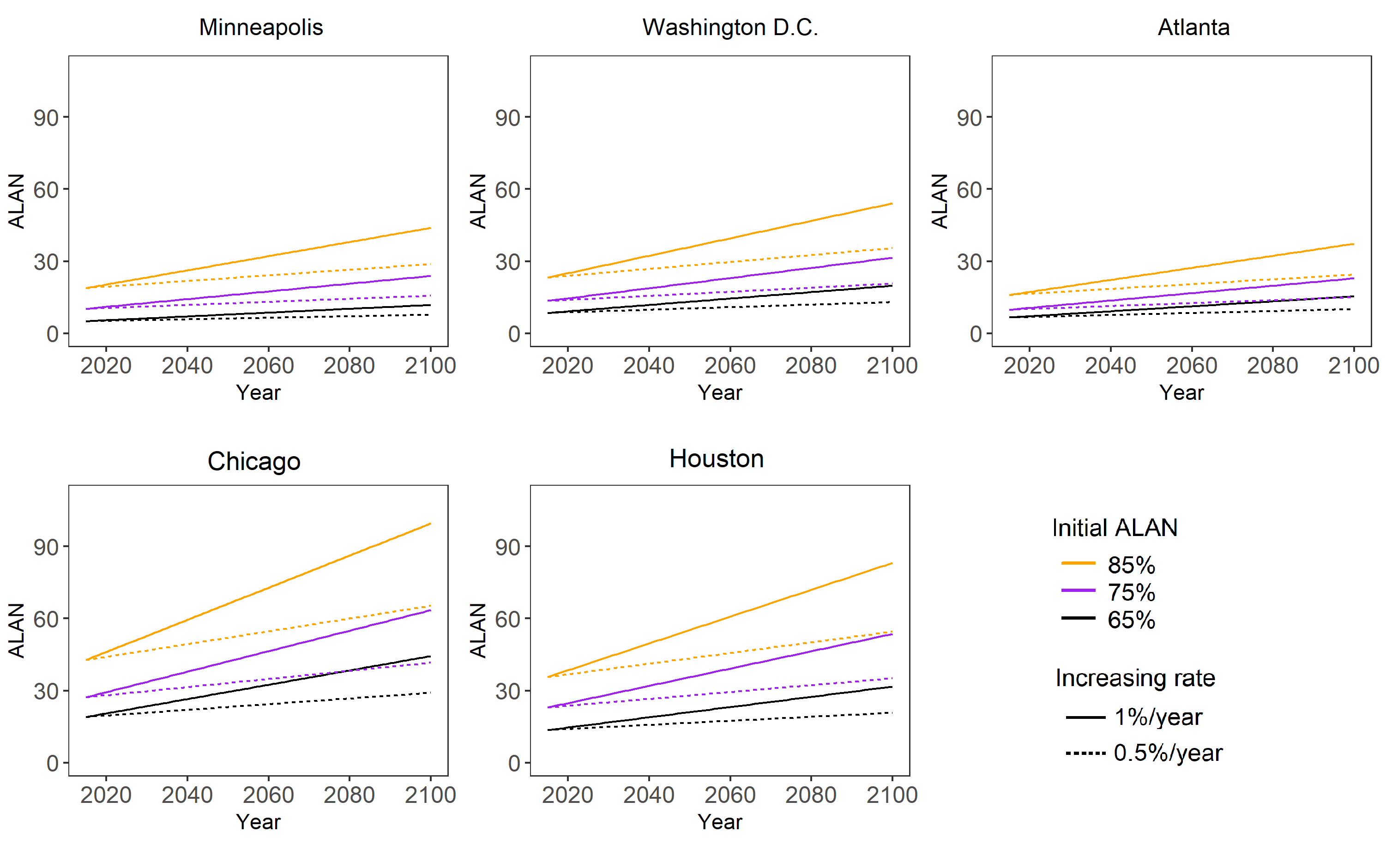


**Fig. S12 Future ALAN increasing scenarios for the five cities.** Three ALAN scenarios (i.e., no increase, 0.5%/year increase, and 1%/year increase) were used in this study, and the latter two increasing scenarios were shown here. The initial ALAN is the percentage quantile of all ALAN grids of each city in 2016.

**Table S1 Summary of the phenological observation and species used in this study.** The number of observations is site-year observations across the conterminous United States during 2011-2016.

| **Phenological stages** | **Species** | **Number of observations** |
| --- | --- | --- |
| Breaking leaf buds | *Acer rubrum* | 553 |
|  | *Acer saccharum* | 224 |
|  | *Betula papyrifera* | 81 |
|  | *Cercis canadensis* | 107 |
|  | *Cornus florida* | 187 |
|  | *Cornus florida-appalachianspring* | 91 |
|  | *Fagus grandifolia* | 148 |
|  | *Forsythia* spp. | 139 |
|  | *Liquidambar styraciflua* | 82 |
|  | *Liriodendron tulipifera* | 146 |
|  | *Populus tremuloides* | 125 |
|  | *Prunus serotina* | 131 |
|  | *Quercus alba* | 135 |
|  | *Quercus lobata* | 85 |
|  | *Quercus rubra* | 183 |
|  | *Syringa chinensis* | 168 |
|  | *Syringa vulgaris* | 367 |
|  | Sum | 2952 |
| Colored leaves | *Acer negundo* | 55 |
|  | *Acer rubrum* | 396 |
|  | *Acer saccharum* | 186 |
|  | *Betula alleghaniensis* | 47 |
|  | *Betula lenta* | 45 |
|  | *Betula papyrifera* | 57 |
|  | *Cercis canadensis* | 62 |
|  | *Cornus florida* | 110 |
|  | *Cornus florida-appalachianspring* | 41 |
|  | *Fagus grandifolia* | 110 |
|  | *Forsythia spp.* | 65 |
|  | *Liquidambar styraciflua* | 85 |
|  | *Liriodendron tulipifera* | 94 |
|  | *Populus deltoides* | 40 |
|  | *Populus tremuloides* | 120 |
|  | *Prunus serotina* | 104 |
|  | *Prunus virginiana* | 55 |
|  | *Quercus alba* | 123 |
|  | *Quercus gambelii* | 52 |
|  | *Quercus macrocarpa* | 41 |
|  | *Quercus rubra* | 163 |
|  | *Tilia americana* | 45 |
|  | *Viburnum lantanoides* | 52 |
|  | Sum | 2148 |

**Table S2 Random effect of species in the linear mixed models.** The random effect column represents changes in phenology that explained by species effect. The negative sign indicates phenology advance in days.

| **Phenological stages** | **Species** | **Random effect** |
| --- | --- | --- |
| Breaking leaf buds | *Acer rubrum* | -0.02 |
|  | *Acer saccharum* | 3.13 |
|  | *Betula papyrifera* | 1.21 |
|  | *Cercis canadensis* | 6.78 |
|  | *Cornus florida* | 0.73 |
|  | *Cornus florida-appalachianspring* | 3.38 |
|  | *Fagus grandifolia* | 8.27 |
|  | *Forsythia* spp. | -4.56 |
|  | *Liquidambar styraciflua* | 5.39 |
|  | *Liriodendron tulipifera* | 1.19 |
|  | *Populus tremuloides* | 4.11 |
|  | *Prunus serotina* | 0.52 |
|  | *Quercus alba* | 7.22 |
|  | *Quercus lobata* | -19.19 |
|  | *Quercus rubra* | 4.74 |
|  | *Syringa chinensis* | -11.52 |
|  | *Syringa vulgaris* | -11.40 |
|  | SD | 7.55 |
| Colored leaves | *Acer negundo* | -6.49 |
|  | *Acer rubrum* | 8.52 |
|  | *Acer saccharum* | 4.75 |
|  | *Betula alleghaniensis* | 1.18 |
|  | *Betula lenta* | -0.95 |
|  | *Betula papyrifera* | -5.97 |
|  | *Cercis canadensis* | -3.52 |
|  | *Cornus florida* | -10.41 |
|  | *Cornus florida-appalachianspring* | 3.87 |
|  | *Fagus grandifolia* | 11.02 |
|  | *Forsythia spp.* | 15.52 |
|  | *Liquidambar styraciflua* | 3.43 |
|  | *Liriodendron tulipifera* | -8.18 |
|  | *Populus deltoides* | -13.62 |
|  | *Populus tremuloides* | 4.47 |
|  | *Prunus serotina* | -6.42 |
|  | *Prunus virginiana* | -13.53 |
|  | *Quercus alba* | 6.71 |
|  | *Quercus gambelii* | 6.18 |
|  | *Quercus macrocarpa* | 1.00 |
|  | *Quercus rubra* | 11.49 |
|  | *Tilia americana* | 0.25 |
|  | *Viburnum lantanoides* | -9.30 |
|  | SD | 8.22 |

**Table S3 Random effect of year in the linear mixed models.** The two columns represent changes in phenology that explained by the year effect. The negative sign indicates phenology advance in days.

| **Year** | **Breaking leaf buds** | **Colored leaves** |
| --- | --- | --- |
| 2011 | 1.26 | 0.76 |
| 2012 | -3.51 | -0.34 |
| 2013 | 1.48 | -0.69 |
| 2014 | 2.07 | -0.06 |
| 2015 | -0.08 | 0.34 |
| 2016 | -1.23 | -0.01 |
| SD | 2.09 | 0.51 |

**Table S4 Summary of the 24 climate models used in this study from the CMIP6 model simulations under Shared Socioeconomic Pathway (SSP) 5-8.5.**

| **Model name** | **Country** | **Variant label** |
| --- | --- | --- |
| AWI-CM-1-1-MR | Germany | r1i1p1f1 |
| BCC-CSM2-MR | China | r1i1p1f1 |
| CAMS-CSM1-0 | China | r1i1p1f1 |
| CanESM5 | Canada | r1i1p1f1 |
| CanESM5-CanOE | Canada | r1i1p2f1 |
| CESM2 | USA | r1i1p1f1 |
| CIESM | China | r1i1p1f1 |
| CMCC-CM2-SR5 | Italy | r1i1p1f1 |
| CNRM-CM6-1 | France | r1i1p1f2 |
| CNRM-CM6-1-HR | France | r1i1p1f2 |
| FGOALS-g3 | China | r1i1p1f1 |
| HadGEM3-GC31-LL | UK | r1i1p1f3 |
| HadGEM3-GC31-MM | UK | r1i1p1f3 |
| IITM-ESM | India | r1i1p1f1 |
| INM-CM4-8 | Russia | r1i1p1f1 |
| INM-CM5-0 | Russia | r1i1p1f1 |
| KACE-1-0-G | South Korea | r1i1p1f1 |
| MIROC6 | Japan | r1i1p1f1 |
| MIROC-ES2H | Japan | r1i1p1f2 |
| MPI-ESM1-2-LR | Germany | r1i1p1f1 |
| MRI-ESM2-0 | Japan | r1i1p1f1 |
| NESM3 | China | r1i1p1f1 |
| NorESM2-LM | Norway | r1i1p1f1 |
| NorESM2-MM | Norway | r1i1p1f1 |

**Table S5 Temperature and ALAN of the five cities studied.** Mean and ± 95% confidence interval are shown for temperature (°C) for 2011-2016. The 65%, 75%, and 85% quantiles of ALAN (nW/cm^2^/sr) were obtained based on all grids with ALAN>0 for each city in 2016.

| **City** | **Spring temperature** | **Summer temperature** | **65% ALAN** | **75% ALAN** | **85% ALAN** |
| --- | --- | --- | --- | --- | --- |
| **Minneapolis** | 7.3 ± 0.4 | 21.0 ± 0.1 | 5.1 | 10.3 | 18.8 |
| **Chicago** | 8.7 ± 0.8 | 21.4 ± 0.4 | 13.6 | 21.4 | 34.6 |
| **Washington D.C.** | 13.0 ± 0.4 | 24.4 ± 0.3 | 8.5 | 13.5 | 23.2 |
| **Atlanta** | 16.0 ± 0.9 | 25.2 ± 0.7 | 6.6 | 9.8 | 16.0 |
| **Houston** | 20.5 ± 1.0 | 28.5 ± 0.4 | 6.1 | 14.4 | 27.6 |
